# Emerging roles of circular RNAs in cancer therapy-induced cardiotoxicity

**DOI:** 10.3389/fcvm.2023.1152436

**Published:** 2023-03-20

**Authors:** Ziji Cheng, Wanting Qin, Shaoling Li, Shuijin Shao, Baonian Liu

**Affiliations:** ^1^ Department of Anatomy, School of Basic Medicine, Shanghai University of Traditional Chinese Medicine, Shanghai, China; ^2^ Department of Pathology, Shanghai Pulmonary Hospital, School of Medicine, Tongji University, Shanghai, China

**Keywords:** biomaker, circRNA, doxorubicin, therapeutic target, cancer therapy-induced cardiotoxicity

## Abstract

Cancer therapy-induced cardiotoxicity (CTIC) is an important cause of death in cancer survivors which often results in the withdrawal or discontinuation of drugs. The underlying mechanisms of CTIC remain unclear. Circular RNAs (circRNAs) are a class of non-coding regulatory RNA molecules which have emerged in recent years. They are generated by back splicing and have powerful biological functions, including transcription and splicing, isolating or building macromolecular scaffolds to interfere with microRNA activity and signaling pathways, and acting as templates for translation. Moreover, circRNAs demonstrate high abundance and significant stability. CircRNAs can be used as novel biomarkers because they often function in a cell-type and tissue-specific manner. CircRNAs have attracted increasing attention in cardiovascular disease research, and recent studies exploring the role of circRNAs in CTIC have had promising results. This review will summarize the current understanding of circRNAs’ biogenesis, regulation and function. Their clinical potential as biomarkers, therapeutic agents and drug targets will also be explored.

## Introduction

The global survival rate of cancer has increased significantly due to improvements in diagnosis and treatment. Unfortunately, cancer treatment is associated with high incidence of adverse cardiac reactions: a rare but fatal condition known as cancer therapy-induced cardiotoxicity (CTIC) (1, 2). CTIC is considered as a cause of death for survivors of breast, prostate and bladder cancer, even surpassing the mortality related to recurrence of the baseline malignancy and impacting the short- and long-term prognosis of patients, which has given rise to the field of onco-cardiology (3). Onco-cardiology focuses on the cardiovascular effects of cancer and its treatment. The most frequently reported cancer CTICs are hypertension, thromboembolism, angiogenesis, QT interval prolongation and heart failure (4, 5). Heart failure caused by CTIC is a growing problem in many cancer survivors. Over the past decade, immunotherapy, particularly immune checkpoint inhibitors (ICIs) and chimeric antigen receptor (CAR) T cell therapy have dramatically changed the landscape of cancer treatment (6). Unfortunately, both ICIs and CAR T cell therapy are also associated with a spectrum of cardiac side effects, such as autoimmune myocarditis, cardiomyopathy, heart failure, etc. (7, 8). CTIC, a side effect of various chemotherapies, targeted anticancer drugs, radiotherapy and immunotherapy, is not yet fully understood. Therefore, a deeper understanding of the molecular mechanisms used to regulate CTIC is required for the development of more effective diagnostics and therapies.

Over the last few decades, as a well-known physiological regulator, non-coding RNAs have been widely explored in the field of cardiovascular research. Increasing evidence shows that circular RNAs (circRNAs) are involved in the biological processes leading to the onset and progression of cardiovascular diseases (9). Many circRNAs have been located in the heart and other organs *via* deep sequencing and new bioinformatics approaches (10–12). Greater breakthroughs have been made in understanding the function of these circRNAs. Previous study has proved that circRNAs can regulate gene expression by modulating transcription and splicing, titrating microRNAs (miRNAs), interacting with proteins and acting as templates for the synthesis of polypeptides (13). The available data suggest that circRNAs may be biomarkers for the diagnosis and/or prognosis of CTIC, as well as acting as potential therapeutic targets. In this review, we summarize the biogenesis, characterization and functions of circRNAs. Then, we outline the possible mechanisms of action of different circRNAs in CTIC and discuss the clinical potential of these circRNAs as biomarkers and therapeutic agents or targets. Finally, we discuss the challenges and biological knowledge gaps which need to be addressed to advance research in this area and bring circRNAs to the forefront of clinical practice in CTIC.

## Biogenesis, characterization and functions of circRNA

Circular RNAs are single-stranded, covalently closed, endogenous biomolecules generated from the precursor mRNAs *via* back-splicing, in which the downstream 5′ splice site is joined to the upstream 3′ splice site in the opposite order across one or more exons (14). Back-splicing of circRNAs formation requires a canonical spliceosome mechanism. Back-splicing occurs both co-transcriptionally and post-transcriptionally (15). Studies have shown that circRNA production is tightly regulated by specific cis-acting elements and trans-acting factors, which have distinct effects on back-splicing (16, 17). Alternative back-splicing and alternative splicing site selection at one gene locus can occur during the formation of circRNAs to form multiple circular RNAs. CircRNAs can be divided into four categories: exonic circRNA, circular intronic RNA, exon-intron circRNA and mitochondria-encoded circRNAs (mecciRNAs) (18, 19). Several exons are contained in most endogenous human circRNAs, usually two or three. Most circRNAs are expressed at low levels compared to the linear RNAs (20). Several researchers have provided evidence that many such circRNAs are caused by mRNA-splicing errors and are nonfunctional (21). Notably, some circRNAs were expressed at higher levels than their corresponding linear mRNAs because their expression was independent of their linear isoforms (22). Recent studies have shown that there are cell type-specific and tissue-specific patterns in circRNAs (23, 24). CircRNAs are significantly enriched in brain and human platelets, as well as during human epithelial–mesenchymal transition and differentiation of hematopoietic progenitors to lymphocytes and bone marrow cells (25, 26). Analysis of non-polyadenylated transcriptomes and RNase R-treated transcriptomes also revealed that the expression of circRNAs is very broad in metazoans, ranging from nematodes, zebrafish, etc. (27–29). Remarkably, once produced, most circRNAs are very stable, as they are not only resistant to degradation caused by the linear RNA decay machinery, but also have a longer half-life compared to their cognate linear RNAs (30). In addition, many circRNAs are expressed in a disease-specific manner, demonstrating extraordinary potential in the pathogenesis of diseases such as tumors, neurodegenerative diseases, etc. (31–34).

Ongoing investigation has revealed that circRNAs can be used as molecular sponges to inhibit microRNA interaction with targets and protein function, or as templates to efficiently generate peptides *via* rolling cycle amplification (35). They can also be used as molecular scaffolds to improve the reaction kinetics of enzyme–substrate interactions (36). Chromatin modifications and facilitation of gene expression were promoted by circRNAs in the nucleus (37). The researchers also revealed that circRNA can mediate cancer metastasis by modulating the alternative splicing of genes (38). CircRNAs are resistant to degradation and play a role in innate immunity, cell proliferation and transformation and neuronal function (39). They can be encapsulated in extracellular vesicles and transported *via* the circulation. Their dysregulation is associated with diseases which are phenotypically analyzed in animal models (40). They are closely associated with urological diseases such as hypertensive nephropathy and diabetic nephropathy (41–43). In addition, some studies found that some circRNAs have anti-cancerous or tumor suppressive effects which allow them to modulate drug resistance in cardiomyopathies (44, 45).

## CircRNA and doxorubicin-induced cardiotoxicity

**Figure 1:**
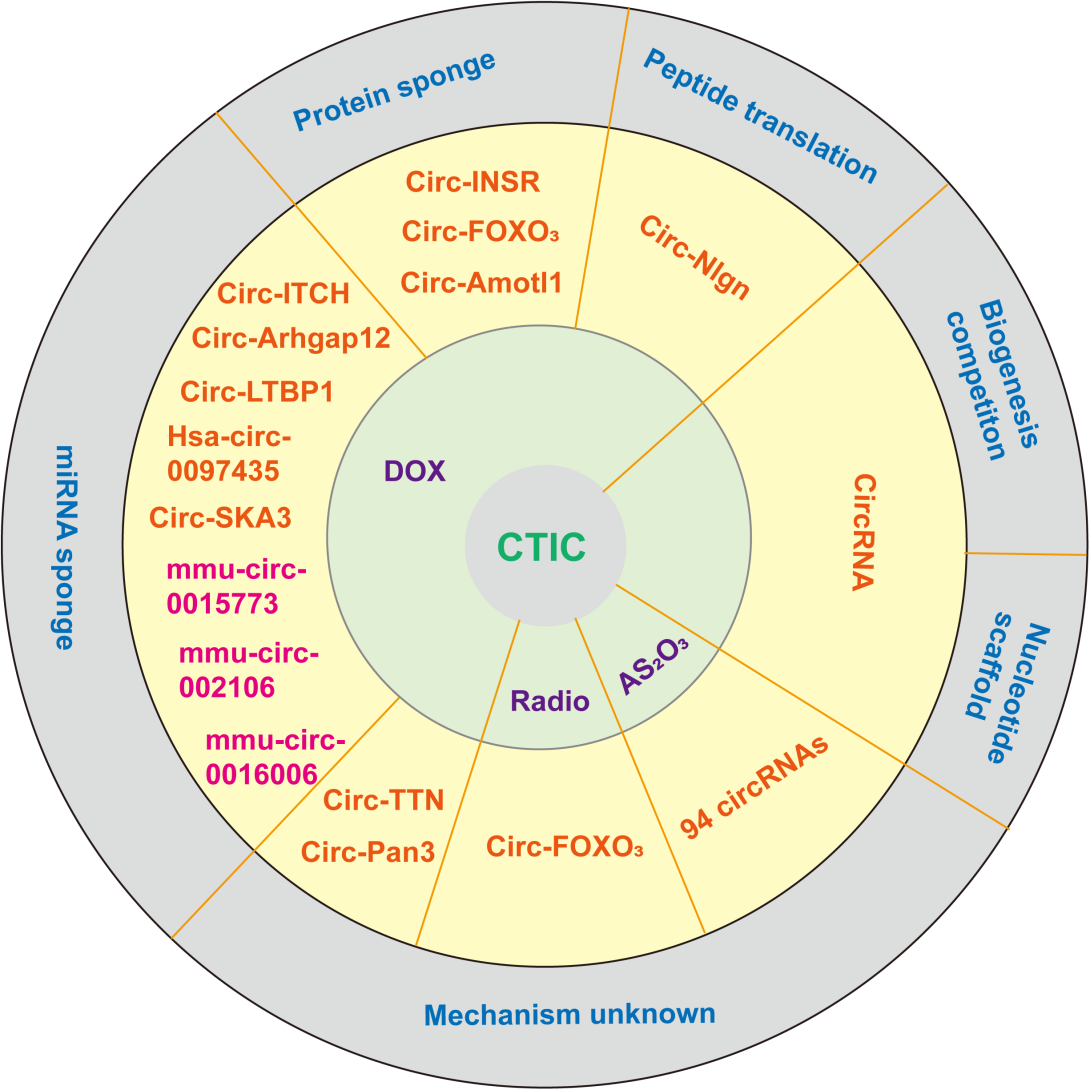
A schematic of different modes of action of circRNAs currently annotated in cells and in CTIC. In addition to pre-mRNA splicing, which can be affected by the interplay between back-splicing and canonical splicing for the same splice site selection, mature circRNAs themselves can act as decoys or sponges for miRNAs and proteins and as RNA scaffolds. A portion of the circRNA can translate peptides. The mechanism of another group of circRNAs is still unclear in CTIC. The pink labeled circular RNAs have potential interaction with miRNA, but further experimental verification is needed to confirm this. miRNA, microRNA; circRNA, circular RNA.

Doxorubicin (DOX) remains the most effective and common chemotherapeutic agent used to treat hematological malignancies and solid tumors (46). However, severe dose-dependent and cumulative cardiotoxicity hampers its clinical use. Over the past few decades, several possible mechanisms of doxorubicin-induced cardiac toxicity (DOXIC) have been proposed by researchers, including overproduction of oxidative stress, DNA damage and altered cardiac energetics, leading to irreversible cell death ultimately (47, 48). However, the question of how to apply DOXIC in treatment has yet to be determined. These circRNAs have not been fully validated and only a few circRNAs have been explored in detail, as shown in Figure 1 and Table 1.

**Table 1:** circRNAs in cancer therapy-induced cardiotoxicity.

CircRNAs	Diseases	Type of experiment	Expression	Mechanisms/Approaches	Function	References
Circ-INSR	Doxorubicin-Induced Cardiotoxicity	*In vitro*/*In vivo*	Down	Interacted with SSBP1 to rescue mitochondrial impairment	DNA damage Apoptosis Mitochondrial dysfunction	Lu et al. (49)
CircITCH	Doxorubicin-Induced Cardiotoxicity	*In vitro*/*In vivo*	Down	Sponged miR-330-5p to upregulate CircITCH/miR-330-5p axis	Oxidative stress DNA damage Apoptosis Regulation of contractility Calcium handling	Han et al. (50)
CircTTN	Doxorubicin-Induced Cardiotoxicity	*In vitro*/*In vivo*	Down	Unknown	Apoptosis	Gupta et al. (51)
Circ-Foxo3	Doxorubicin-Induced Cardiotoxicity	*In vitro*/*In vivo*	Up	Unknown	DNA damage Apoptosis Oxidative stress	Du et al. (52)
CircNlgn	Doxorubicin-Induced Cardiotoxicity	*In vivo*	Down	Interacted with H2AX to upregulate IL-1b, IL-2Rb, IL-6, EGR1 and EGR3	Proliferation, Survival Apoptosis	Xu et al. (53)
CircArhgap12	Doxorubicin-Induced Cardiotoxicity	*In vivo*	Up	Sponged miR-135a-5p	Apoptosis Oxidative stress	Wang et al. (54)
CircPan3	Doxorubicin-Induced Cardiotoxicity	*In vitro*/*In vivo*	Down	Unknown	Myocardial fibrosis Apoptosis	Ji et al. (55)
Circ-Amotl1	Doxorubicin-Induced Cardiotoxicity	*In vitro*/*In vivo*	Down	Interacted with PDK1 and AKT1 to facilitate AKT activation and nuclear translocation	Proliferation Survival Apoptosis	Zeng et al. (56)
Hsa_circ_0097435	Doxorubicin-Induced Cardiotoxicity	*In vivo*	Up	Sponged multiple microRNAs to regulate the expression of downstream target genes of miRNA	Apoptosis	Han et al. (57)
Circ-LTBP1	Doxorubicin-Induced Cardiotoxicity	*In vivo*	Up	Sponged miR-107 to elevate ADCY1	Proliferation Inflammation Apoptosis Oxidative stress	Li et al. (58)
Circ-SKA3	Doxorubicin-Induced Cardiotoxicity	*In vitro*	Up	Sponged miR-1303 to upregulate TLR4	Apoptosis	Li et al. (59)
mmu_circ_0015773, mmu_circ_0002106, mmu_circ_0016006	Doxorubicin-Induced Cardiotoxicity	*In vitro*	Up	Sponged miR-344g-3p and miR-22-3p to regulate the expression of mRNA	Apoptosis	Xing et al. (60)
The ATO-related lncRNA- circRNA co-regulation network	Arsenic Trioxide- Induced Cardiotoxicity	*In vivo*	down	Interacted with protein-coding genes and/or regulated miRNA.	Apoptosis Inflammation	Jiang et al. (61)
CircFOXO3	Radiation-Induced Cardiotoxicity	*In vitro*	Up	Unknown	DNA damage, Apoptosis	Qiu et al. (62)

## Circ-INSR

Circ-INSR is derived from the host gene encoding the insulin receptor (INSR). It is a conserved non-coding RNA. In a recent issue of *European Heart Journal*, Lu et al. studied the role of Circ-INSR in DOXIC (49). They found that circ-INSR significantly decreased in patients suffering from cardiotoxicity after the DOX therapy and in HL-1 cardiomyocyte-like cells and human-induced pluripotent stem-cell-derived cardiomyocytes after DOX treatment. *In vitro*, siRNA-mediated circ-INSR down-regulation in cardiomyocytes leads to impaired metabolic activity and induces apoptosis during DOX stress. Overexpression of circ-INSR by AAV6 (adeno-associated virus serotype 6) in HL-1 cardiomyocytes and primary rat cardiomyocytes attenuated DOX-induced cardiomyocyte injury. *In vivo*, overexpression of circ-INSR by AAV6 significantly improved DOX-induced cardiac atrophy and dysfunction. In addition, cardiomyocyte death under DOX stress was reduced by Circ-INSR mimics. Furthermore, the expression of Circ-INSR was found to be regulated by breast cancer type 1 susceptibility protein, while Circ-INSR physically interacts with single-stranded DNA-binding protein 1 to regulate apoptotic and metabolic pathways in cardiomyocytes. In summary, these results illustrate the promising development of Circ-INSR in preventing anti-cancer drug-induced cardiotoxicity.

## CircITCH

CircITCH is a tumor suppressor whose host gene is ITCH (E3 ubiquitin protein ligase). A further investigation reported in *Circulation Research* showed that CircITCH also plays a role in DOXIC (50). First, the authors performed circular RNA sequencing to identify CircITCH involved in DOXI pathogenesis. They found that pluripotent stem cell-derived cardiomyocytes (hiPSC-CMs) were induced in DOX-treated humans and CircITCH was downregulated in autopsy specimens from cancer patients with DOX-induced cardiomyopathy. DOX-induced hiPSC-CM injury and dysfunction was prevented by the overexpression of CircITCH. DOX-induced hiPSC-CM cellular/mitochondrial oxidative stress and DNA damage were regulated. Mechanistically, miR-330-5p was identified as a direct target of CircITCH. The indicators in quantitative polymerase chain reaction were upregulated by CircITCH which serve as an endogenous miR-330-5p sponge. The interaction between miR-330-5p and 3’-UTRs of SIRT6, BIRC5 and ATP2A2 mRNA alleviated CircITCH-mediated DOXIC. Finally, overexpression of an AAV9 (adeno-associated virus serotype 9) vector based on the highly conserved CircITCH partially prevented DOXIC in mice. Collectively, these methods validated the role of CircITCH in DOXIC progression and identified it as a meaningful therapeutic agent for DOX-based chemotherapy.

## CircTTN

CircTTN is derived from the titin (TTN) gene, which produces the largest protein in humans and plays an important role in cardiac homeostasis. The production of a large proportion of circTTNs depends on the splice regulator RBM20 (63). CircTTN has multiple functions and plays an important role in cardiac homeostasis (64). The role of Quaking (QKI) was explored by Gupta et al. in an issue of *Circulation Research* (51). QKI is an RBP which plays an important role in DOX-induced heart failure. AAV9-mediated cardiac overexpression of QKI isoform Qki5 attenuated DOX-induced cardiac apoptosis, atrophy and improved cardiac function. Moreover, Qki5 was associated with expression of circular RNAs derived from Ttn, Fhod3 and Strn3. Furthermore, siRNA-mediated inhibition of CircTTN 105–111 increased susceptibility to DOX. The knockdown of CircTTN 105–111 in pLV Qki5-overexpressing cell line also resulted in induced caspase activity. On the contrary, lentiviral mediated overexpression of CircTTN 105–111 led to lower caspase 3/7 activity and increased cell survival. This suggests that circular RNAs, especially CircTTN 105–111, are downstream mediators of Qki5-mediated protective effects. However, the roles of the other circRNA candidates in DOXIC and the functional relationship between these circRNAs, especially TTN 105–111, and QKI-5 has not been established. In addition, the potential mechanisms associated with the inhibition of apoptosis in cardiac myocytes by CircTTN 105–111 have yet to be studied. Despite the limitations described above, this study may provide a rational basis for new therapeutic strategies for DOXIC.

## Circ-Foxo3

CircRNA Forkhead box O3 (Circ-Foxo3) formed from the exon 2 of FOXO3 plays important roles in cancer (65). The role of circ-Foxo3 in cardiovascular disease has gradually attracted attention. In a previous issue of *European Heart Journal*, Du et al. explored the potential effect of circ-Foxo3 on DOX-induced cardiomyopathy (52). They found that DOX treatment induced mouse heart senescence and increased the circ-Foxo3 level. Overexpression of circ-Foxo3 enhanced DOX-induced mouse heart senescence, apoptosis and cardiac fibrosis, which were repressed by circ-Foxo3 silencing. Functionally, cardiac function deterioration promoted ectopic expression of circ-Foxo3; the process appeared to be reduced by silencing endogenous circ-Foxo3. In brief, CircFoxo3 is thought to prevent the localization of FAK to mitochondria or the translocation of hypoxia-inducible factor 1α (HIF1α) to the nucleus in stressed cells through the interaction of senescence-associated proteins ID-1 and E2F1, as well as stress-associated proteins FAK and HIF1α in the cytoplasm.

## CircNlgn

Circular neuroligin RNA (circNlgn) produced from the neuroligin gene. CircNlgn is involved in cardiac overload-induced remodeling of fibrosis and heart failure through its translation protein Nlgn173 (66). Recently, the research team investigated the role of the circNlgn in DOX-induced cardiac remodeling and fibrosis (53). A transgenic mouse line overexpressing circNlgn was developed. CircNlgn was found to be a mediator of DOX-induced cardiac fibrosis, while cardiac function decreased as a result of the increased expression of circNlgn. Myocardial fibrosis was induced by upregulation of Gadd45b, Sema4C and RAD50 and activation of p38 and p-JNK in circNlgn transgenic hearts. The effect of DOX on cardiac cell activity was reduced by small interfering RNA (siRNA) silencing targeting circNlgn and the expression of DOX-induced fibrosis-related molecules was thus prevented. H2AX can be bound and activated by circNlgn-translated protein (Nlgn173). γH2AX was produced and IL-1β, IL-2Rb, IL-6, EGR1, and EGR3 were produced during this process. The researchers determined that DOX-induced prevention of cardiomyocyte apoptosis, an increase in cardiomyocyte viability, a decrease in cardiac fibroblast proliferation, and inhibition of collagen production could be achieved by silencing these molecules. This mechanism may have therapeutic significance in alleviating the side effects of DOX in cancer patients.

## CircArhgap12

A study by Wang et al. explored the regulatory role of differentially expressed circRNAs in mouse cardiomyocytes in DOX-induced cardiotoxicity (54). Expression profiles of circRNAs were obtained by next-generation sequencing and 48 significantly upregulated and 16 downregulated circRNAs were found in the DOX-injected group. Bioinformatics analysis such as KEGG and KOG classifications revealed that the differentially expressed circRNAs may be related to DOX-induced cardiomyocyte apoptosis. Furthermore, they proved that circRNA encoded by the Arhgap12 gene (known as circArhgap12) was upregulated in mouse heart tissue after DOX intervention. Both RNA sequencing results and subsequent RT-qPCR validation indicated that circArhgap12 coded by the Arhgap12 gene was upregulated in the mouse heart tissue following DOX intervention. DOX-induced cardiomyocyte apoptosis and oxidative stress were aggravated by circArhgap12 sponging miR-135a-5p. Further experimental results proved ADCY1 was a potential target of miR-135a-5p through the use of Cytoscape and luciferase reporter assays. However, the functional relationship between miR-135a-5p and ADCY1 has not been established and the role and underlying mechanism of ADCY1 in DOXIC have yet to be determined. The interactions in the circRNA-miRNA-mRNA network may be an important mechanism used to regulate DOXIC. In summary, this study explored the use of circRNA network-based therapy for the modulation of DOXIC.

## CircPan3

Circular Pan3 (circPan3) derived from the poly(A) specific ribonuclease subunit PAN3 (Pan3) gene has been proved to positively contribute to cardiovascular disease (67). Ji et al. explored the mechanism of DOXIC (67) and showed that circPan3 was negatively regulated in cardiomyocytes by DOX treatment. The overexpression of circPan3 demonstrated a protective effect against DOX-induced myocardial apoptosis. Moreover, this study revealed the upstream signaling molecular mechanism regulating circPan3, in which QKI directly upregulates circPan3. As a negative regulator of circPan3, miR-31-5p worked by suppressing QKI directly. In conclusion, the researchers found that silencing of QKI using miR-31-5p can contribute to the downregulation of circPan3 by DOX, which may be a potential therapeutic direction for DOXIC.

## Circ-Amotl1

In a recent issue of *Theranostics*, Zeng et al. described a circular RNA, circ-Amotl1, derived from angiomotin-like 1 gene which is highly expressed in neonatal human cardiac tissue and explored the effect of circ-Amotl1 on DOXIC in mice (56). They showed that ectopic expression of circ-Amotl1 improved DOX-induced cardiac dysfunction and relieved the cumulative effects of DOX. In addition, HE and Sirius-Red staining showed that the enlarged left ventricle and remodeling of the LV were reduced by ectopic expression of circ-Amotl1. Moreover, TUNEL staining showed that DOX-induced cardiomyocytes apoptosis was abolished by circ-Amotl1 delivery. Mechanistically, the researchers revealed that myocardial protective nuclear translocation of p-AKT was facilitated by circ-Amotl1 binding to PDK1 and AKT1 simultaneously. In conclusion, these results suggested the potential of circ-Amotl1 expression for a therapeutic approach to prevent adverse cardiac remodeling.

## Hsa_circ_0097435

In an issue of *Frontiers in Genetics*, Han et al. screened for the circular RNA (hsa_circ_0097435), which was significantly expressed in patients with heart failure, and explored its effect on DOX-induced apoptosis in human cardiomyocyte-like AC16 cells (57). They showed that the expression level of hsa_circ_0097435 in AC16 cells was time-dependently upregulated by DOX. The overexpression of hsa_circ_0097435 promoted cardiomyocyte apoptosis, while silencing hsa_circ_0097435 inhibited apoptosis. The results showed that hsa_circ_0097435 plays a key role in the pathogenesis and development of HF. Therefore, hsa_circ_0097435 was proved to be a blood biomarker that provides a strong basis for the development of new therapies for heart failure.

## Circ-LTBP1

Circ-LTBP1 is encoded by latent transforming growth factor (TGF)-beta binding protein-1 (LTBP1). Li et al. explored the roles of circ-LTBP1 in DOXIC (58). Circ-LTBP1 was overexpressed in DOX-stimulated AC16 cells. Effects on AC16 cell proliferation, inflammation, apoptosis and oxidative stress could be abolished by si-circ-LTBP1 inhibition of Circ-LTBP1. Furthermore, circ-LTBP1 negatively regulated expression of miR-107 and performed its function by sponging miR-107 in DOX-stimulated AC16 cells. The researchers also revealed that miR-107 exerted a cardioprotective effect on DOX-induced AC16 cells through ADCY1. Overall, these results indicate that circ-LTBP1 participates in DOXIC by targeting the miR-107/ADCY1 signaling pathway, which provides biomarkers and potential targets.

## Circ-SKA3

CircRNAs spindle and exciton-associated protein 3 (circ-SKA3) facilitate the malignant biological behaviors of tumors. In a recent study, Li et al. investigated the role and mechanism of circ-SKA3 in DOXIC (59). They showed that circ-SKA3 was upregulated in DOX-treated AC16 cells and the knockdown of circ-SKA3 protected AC16 cells from DOXIC *via* the miR-1303/TLR4 axis. In addition, they stated that circ-SKA3 can be packaged into exosomes and the exosomal circ-SKA3 level was elevated in DOX-treated AC16 cells, which indicated that exosomal circ-SKA3 might be an ideal biomarker for preventing DOXIC.

## Other circRNAs

A study by Xing et al. (60), reported in the *Journal of Applied Toxicology*, comprehensively analyzed the expression profiles of circular RNA and mRNA in DOXIC mice. Eleven significantly altered circRNAs were identified *via* whole transcriptome sequencing in the DOXIC mouse heart, of which seven were upregulated and four were downregulated. Dysregulation of three circRNAs (mmu_circ_0015773, mmu_circ_0002106 and mmu_circ_0016006) were verified by real-time quantitative PCR, and 35 DEmRNAs were discovered using the ceRNA network. Further bioinformatics analysis showed that the apoptotic process of cardiomyocytes was activated by the alterations in three circRNAs. In summary, this study provides new ideas about the mechanism of DOX-induced cardiotoxicity and suggests potential biomarkers or therapeutic targets.

## CircRNA and arsenic trioxide-induced cardiotoxicity

Arsenic trioxide (As_2_O_3_, ATO), a traditional Chinese medicine, is emerging as a frontline antineoplastic agent for the treatment of acute promyelocytic leukemia (68). Unfortunately, the increased use of ATO has resulted in adverse cardiac effects, the main manifestations of which are long QT syndrome, tachycardia and sudden cardiac death (69). Cardiotoxicity poses a major obstacle to ATO's therapeutic value and its mechanism of cardiotoxicity has not been fully explored. Previous studies found that ATO induces oxidative stress-mediated cardiotoxicity by causing apoptosis and imbalance in the redox state of cardiomyocytes through the activation pathway of caspase-3 or changes in internal MMP (70). In addition, the imbalance of trace elements caused by arsenic trioxide may lead to an imbalance in mitochondrial dynamics, which leads to the induction of apoptosis and limits cellular metabolism. In a recent issue of *Biomedicine and Pharmacotherapy*, Jiang et al. attempted to explain the molecular basis of ATO-induced cardiotoxicity from a transcriptome analysis and the circRNA-lncRNA network construction in ATO-treated mice myocardium (61). They observed 94 differentially expressed circRNAs, 49 upregulated and 45 downregulated, in the myocardium of ATO-treated mice. The expression of mm9_circ_009519 (upregulated) and mm9_circ_016007 (downregulated) validated by real-time PCR was in accordance with the RNA sequencing results. Moreover, a circRNA-mRNA co-expression network containing 94 circRNAs and 113 mRNA was constructed to explain the functions of aberrantly expressed circRNAs. Nine circRNAs, five miRNAs and eight mRNAs were constructed by a circRNA-miRNA-mRNA network. Thus, these aberrantly expressed circRNA may be involved in ATO-induced cardiotoxicity by interacting with protein-coding genes and/or regulating miRNA to perform biological functions. Therefore, this study provides a novel strategy for the prevention and treatment of ATO-induced cardiotoxicity.

## CircRNA and radiation-induced cardiotoxicity

Radiation therapy is one of the main treatment options for cancer, which can lead to delayed heart damage. However, the underlying mechanisms and biomarkers of radiation-induced cardiotoxicity are not yet clear. Qiu et al. explored the role of cirFOXO3 in radiation-induced cardiotoxicity and showed that circFOXO3 is significantly upregulated in cardiomyocytes after radiation (62). In addition, they established circFOXO3-knockdown or -overexpression cardiomyocytes and showed that knockdown of the circFOXO3 aggregated radiation-induced DNA damage and apoptosis in cardiomyocytes, which were reversed by circFOXO3 overexpression.

Mechanistically, they found that the levels of pro-apoptotic markers Bax, caspase 3 and caspase 7 were elevated by circFOXO3 and anti-apoptotic marker Bcl-2 expression was decreased. Thus, they summarized that circFOXO3 protected cardiomyocytes from radiation-induced cardiotoxicity by reducing DNA damage and apoptosis. CircFOXO3 is expect to be a potential target for the treatment of radiation cardiotoxicity.

## Conclusions and perspectives

Cancers pose a serious threat to the global economy and human health, though long-term survival rates have improved in recent years as a result of various new anti-cancer therapies. Unfortunately, the risk of cardiovascular complications has also increased due to the adverse side effects of these treatment methods. Previous studies have found that circRNAs perform important functions in a variety of human diseases, particularly cardiovascular disease (71). A brief overview of the discovery, biogenesis and general function of circRNAs was summarized in this review. Then, we described relevant studies that highlight circRNAs’ potential as therapeutic targets for cardiotoxicity and explained the hypothesized mechanisms of their biological action, revealing the therapeutic and diagnostic potential of circRNAs.

RNA therapy is a rapidly evolving class of drugs and therapies, which is transitioning from basic to clinical practice at unprecedented speed (72). CircRNA-based therapeutic approaches have promising medical and research applications in therapeutic drugs and protein replacement therapies for prophylactic vaccines (73). On the one hand, circRNA therapy, like other RNA therapies, has emerged as a potential therapy which can modulate gene expression or exert modular effects (74). On the other hand, it uses other methods, such as CRISPR-Cas9 or siRNA, to target local circRNAs for biomarker or sponging treatment of different diseases (75, 76). Emerging data suggest that circRNAs have exceptional promise as therapeutic agents, as well as potential biomarkers for CTIC. However, only a few circRNAs are involved in CTIC. CircRNA has no applicability in clinical practice. At present, several problems hinder the translation prospects of circRNAs. Firstly, the cyclic nature of circRNAs leads to specific technical problems in detection and quantification, which may reduce the reproducibility and complicate the development of the biomarker. Except for the BSJ site, the primary sequence of circRNA is identical to its homologous linear RNA. Thus, distinguishing circRNAs from their linear isoforms remains challenging at multiple levels, such as annotation, validation, etc. Secondly, since the expression level of circRNAs is generally low in mammals and binding sites with miRNAs are relatively few in number, the idea of using circRNAs to control the stability and quantity of miRNAs and achieve measurable effects should be considered carefully. However, because of the low abundance of circRNAs, the probe must be restricted to the region spanning the BSJ. Other barriers include specific conformations of the BSJ and interacting proteins which may affect the sensitivity of the probe; thus, it is difficult to detect circRNAs by using Fluorescence *In Situ* Hybridization (FISH). Large-scale discovery and validation studies must be conducted before circRNAs can be widely accepted as molecules with clinical diagnostic and therapeutic value. Thirdly, the specificity, sensitivity, and stability of the circRNAs under investigation as potential pathological biomarkers requires further careful evaluation. Fourthly, the consistency of the computational tools used in circRNAs studies is found to be relatively low. There is currently no systematic method for identifying circRNAs in the human transcriptome. Fifthly, the problems of controlling the expression level of circRNA to avoid sustained overexpression due to its exceptional stability, large-scale manufacturing of highly purified artificial circRNAs and targeted delivery of circRNAs still need to be resolved in circRNA-based therapeutic approaches. Finally, research on circRNA biology is developing rapidly, but its studies exploring combination with CTIC are lagging. At present, the circRNAs related to CTIC reported in this review are basically exonic circRNAs. Most of them function in the cytoplasm. Therefore, the process of circRNA nuclear export is crucial for its function. Study has shown that efficient circRNA nuclear export is a previously unappreciated layer of regulation which is critical to normal physiological function (77). The abnormalities in circRNA nuclear export lead to physiological defects (78, 79). No studies have been reported concerning the export of circRNAs in CTIC. Therefore, these challenges may be addressed and overcome through further research.

In summary, we have reviewed recent findings which illustrate the important role of circRNAs as therapeutic targets and biomarkers in CTIC. These studies provide new insights into the future of diagnosis and personalized medicine for CTIC patients. Future research is needed to explore their potential as therapeutic agents in animal models. Promising biomarker candidates should be validated in multicenter trials. With the deepening of circRNA research, the field of circRNA research has great translational prospects.

## References

[1] Devaux Y Creemers EE Boon RA Werfel S Thum T Engelhardt S Cardiolinc network. Circular RNAs in heart failure. Eur J Heart Fail. (2017) 19:701–9. 10.1002/ejhf.801 28345158

[2] Minotti G Menna P Salvatorelli E Cairo G Gianni L. Anthracyclines: molecular advances and pharmacologic developments in antitumor activity and cardiotoxicity. Pharmacol Rev. (2004) 56:185–229. 10.1124/pr.56.2.6 15169927

[3] Bisceglia I Cartoni D Petrolati S. Concepts in cardiac oncology. Eur Heart J Suppl. (2020) 22:L19–23. 10.1093/eurheartj/suaa127 33654463 PMC7904054

[4] Yeh ET Bickford CL. Cardiovascular complications of cancer therapy: incidence, pathogenesis, diagnosis, and management. J Am Coll Cardiol. (2009) 53:2231–47. 10.1016/j.jacc.2009.02.050 19520246

[5] Darby SC Cutter DJ Boerma M Constine LS Fajardo LF Kodama K Radiation-related heart disease: current knowledge and future prospects. Int J Radiat Oncol Biol Phys. (2010) 76:656–65. 10.1016/j.ijrobp.2009.09.064 20159360 PMC3910096

[6] Dal'bo N Patel R Parikh R Shah SP Guha A Dani SS Cardiotoxicity of contemporary anticancer immunotherapy. Curr Treat Options Cardiovasc Med. (2020) 22:62. 10.1007/s11936-020-00867-1 33162729 PMC7605901

[7] Heinzerling L Ott PA Hodi FS Husain AN Tajmir-Riahi A Tawbi H Cardiotoxicity associated with CTLA4 and PD1 blocking immunotherapy. J Immunother Cancer. (2016) 4:50. 10.1186/s40425-016-0152-y 27532025 PMC4986340

[8] Rahouma M Karim NA Baudo M Yahia M Kamel M Eldessouki I Cardiotoxicity with immune system targeting drugs: a meta-analysis of anti-PD/PD-L1 immunotherapy randomized clinical trials. Immunotherapy. (2019) 8:725–73. 10.2217/imt-2018-0118 31088241

[9] Wang L Meng X Li G Zhou Q Xiao J. Circular RNAs in cardiovascular diseases. Adv Exp Med Biol. (2018) 1087:191–204. 10.1007/978-981-13-1426-1_15 30259367

[10] Memczak S Jens M Elefsinioti A Torti F Krueger J Rybak A Circular RNAs are a large class of animal RNAs with regulatory potency. Nature. (2013) 495:333–8. 10.1038/nature11928 23446348

[11] Hu W Han Q Zhao L Wang L. Circular RNA circRNA_15698 aggravates the extracellular matrix of diabetic nephropathy mesangial cells via miR-185/TGF-β1. Cell Physiol. (2019) 234:1469–76. 10.1002/jcp.26959 30054916

[12] Hansen TB Venø MT Damgaard CK Kjems J. Comparison of circular RNA prediction tools. Nucleic Acids Res. (2016) 44:e58. 10.1093/nar/gkv1458 26657634 PMC4824091

[13] Wu N Qadir J Yang BB. CircRNA perspective: new strategies for RNA therapy. Trends Mol Med. (2022) 28:343–4. 10.1016/j.molmed.2022.02.002 35232670

[14] Barrett SP Salzman J. Circular RNAs: analysis, expression and potential functions. Development. (2016) 143:1838–47. 10.1242/dev.128074 27246710 PMC4920157

[15] Zhang Y Xue W Li X Zhang J Chen S Zhang JL The biogenesis of nascent circular RNAs. Cell Rep. (2016) 15:611–24. 10.1016/j.celrep.2016.03.058 27068474

[16] Jeck WR Sorrentino JA Wang K Slevin MK Burd CE Liu J Circular RNAs are abundant, conserved, and associated with ALU repeats. RNA. (2013) 19:141–57. 10.1261/rna.035667.112 23249747 PMC3543092

[17] Li X Yang L Chen LL. The biogenesis, functions, and challenges of circular RNAs. Mol Cell. (2018) 71:428–42. 10.1016/j.molcel.2018.06.034 30057200

[18] Wang F Nazarali AJ Ji S. Circular RNAs as potential biomarkers for cancer diagnosis and therapy. Am J Cancer Res. (2016) 6:1167–76. PMID: 27429839; PMCID: PMC4937728 27429839 PMC4937728

[19] Liu X Wang X Li J Hu S Deng Y Yin H Identification of mecciRNAs and their roles in the mitochondrial entry of proteins. Sci China Life Sci. (2020) 10:1429–49. 10.1007/s11427-020-1631-9 32048164

[20] Salzman J Chen RE Olsen MN Wang PL Brown PO. Cell-type specific features of circular RNA expression. PLoS Genet. (2013) 9:e1003777. 10.1371/journal.pgen.1003777 24039610 PMC3764148

[21] Xu C Zhang J. Mammalian circular RNAs result largely from splicing errors. Cell Rep. (2021) 64:109439. 10.1016/j.celrep.2021.109439 PMC8365531 34320353

[22] Westholm JO Miura P Olson S Shenker S Joseph B Sanfilippo P Genome-wide analysis of drosophila circular RNAs reveals their structural and sequence properties and age-dependent neural accumulation. Cell Rep. (2014) 9:1966–80. 10.1016/j.celrep.2014.10.062 25544350 PMC4279448

[23] Xia S Feng J Lei L Hu J Xia L Wang J Comprehensive characterization of tissue-specific circular RNAs in the human and mouse genomes. Brief Bioinform. (2017) 18:984–92. 10.1093/bib/bbw081 27543790

[24] Zhou C Molinie B Daneshvar K Pondick JV Wang J Genome-wide maps of m6A circRNAs identify widespread and cell-type-specific methylation patterns that are distinct from mRNAs. Cell Rep. (2017) 20:2262–76. 10.1016/j.celrep.2017.08.027 28854373 PMC5705222

[25] Rybak-Wolf A Stottmeister C Glažar P Jens M Pino N Giusti S Circular RNAs in the mammalian brain are highly abundant, conserved, and dynamically expressed. Mol Cell. (2015) 58:870–85. 10.1016/j.molcel.2015.03.027 25921068

[26] Nicolet BP Engels S Aglialoro F van den Akker E von Lindern M Wolkers MC. Circular RNA expression in human hematopoietic cells is widespread and cell-type specific. Nucleic Acids Res. (2018) 46:8168–80. 10.1093/nar/gky721 30124921 PMC6144802

[27] Venø MT Hansen TB Venø ST Clausen BH Grebing M Finsen B Spatio-temporal regulation of circular RNA expression during porcine embryonic brain development. Genome Biol. (2015) 16:245. 10.1186/s13059-015-0801-3 26541409 PMC4635978

[28] Abdelmohsen K Panda AC De S Grammatikakis I Kim J Ding J Circular RNAs in monkey muscle: age-dependent changes. Aging. (2015) 7:903–10. 10.18632/aging.100834 26546448 PMC4694061

[29] Sanger HL Klotz G Riesner D Gross HJ Kleinschmidt AK. Viroids are single-stranded covalently closed circular RNA molecules existing as highly base-paired rod-like structures. Proc Natl Acad Sci USA. (1976) 73:3852–6. 10.1073/pnas.73.11.3852 1069269 PMC431239

[30] Enuka Y Lauriola M Feldman ME Sas-Chen A Ulitsky I Yarden Y. Circular RNAs are long-lived and display only minimal early alterations in response to a growth factor. Nucleic Acids Res. (2016) 44:1370–83. 10.1093/nar/gkv1367 26657629 PMC4756822

[31] Kumar L Shamsuzzama Haque R Baghel T Nazir A. Circular RNAs: the emerging class of non-coding RNAs and their potential role in human neurodegenerative diseases. Mol Neurobiol. (2017) 54:7224–34. 10.1007/s12035-016-0213-8 27796758

[32] Uchida S Dimmeler S. Long noncoding RNAs in cardiovascular diseases. Circ Res. (2015) 116:737–50. 10.1161/CIRCRESAHA.116.302521 25677520

[33] Esteller M. Non-coding RNAs in human disease. Nat Rev Genet. (2011) 12:861–74. 10.1038/nrg3074 22094949

[34] Aufiero S van den Hoogenhof MMG Reckman YJ Beqqali A van der Made I Kluin J Cardiac circRNAs arise mainly from constitutive exons rather than alternatively spliced exons. RNA. (2018) 24:815–27. 10.1261/rna.064394.117 29567830 PMC5959250

[35] Kristensen LS Andersen MS Stagsted LVW Ebbesen KK Hansen TB Kjems J. The biogenesis, biology and characterization of circular RNAs. Nat Rev Genet. (2019) 20:675–91. 10.1038/s41576-019-0158-7 31395983

[36] Spitale RC Tsai MC Chang HY. RNA templating the epigenome: long noncoding RNAs as molecular scaffolds. Epigenetics. (2011) 6:539–43. 10.4161/epi.6.5.15221 21393997 PMC3230545

[37] Chen N Zhao G Yan X Lv Z Yin H Zhang S A novel FLI1 exonic circular RNA promotes metastasis in breast cancer by coordinately regulating TET1 and DNMT1. Genome Biol. (2018) 19:218. 10.1186/s13059-018-1594-y 30537986 PMC6290540

[38] Wang X Li J Bian X Wu C Hua J Chang S CircURI1 interacts with hnRNPM to inhibit metastasis by modulating alternative splicing in gastric cancer. Proc Natl Acad Sci U S A. (2021) 118:e2012881118. 10.1073/pnas.2012881118 34385309 PMC8379983

[39] Liu CX Li X Nan F Jiang S Gao X Guo SK Structure and degradation of circular RNAs regulate PKR activation in innate immunity. Cell. (2019) 1774:865–80. 10.1016/j.cell.2019.03.046 31031002

[40] Liu CX Chen LL. Circular RNAs: characterization, cellular roles, and applications. Cell. (2022) 185:2016–34. 10.1016/j.cell.2022.04.021 35584701

[41] Guo G Wang H Ye L Shi X Yan K Lin K Hsa_circ_0000479 as a novel diagnostic biomarker of systemic lupus erythematosus. Front Immunol. (2019) 10:2281. 10.3389/fimmu.2019.02281 31608065 PMC6771011

[42] Cao Y Mi X Zhang D Wang Z Zuo Y Tang W. Transcriptome sequencing of circular RNA reveals a novel circular RNA-has_circ_0114427 in the regulation of inflammation in acute kidney injury. Clin Sci. (2020) 134:139–54. 10.1042/CS20190990 31930399

[43] McCullough PA. Why is chronic kidney disease the “spoiler” for cardiovascular outcomes?. J Am Coll Cardiol. (2003) 41:725–8. 10.1016/S0735-1097(02)02955-8 12628713

[44] Zhou B Zheng P Li Z Li H Wang X Shi Z CircPCNXL2 sponges miR-153 to promote the proliferation and invasion of renal cancer cells through upregulating ZEB2. Cell Cycle. (2018) 17:2644–54. 10.1080/15384101.2018.1553354 30488762 PMC6300113

[45] Zhang D Yang XJ Luo QD Fu DL Li ZL Zhang P Down-regulation of circular RNA_000926 attenuates renal cell carcinoma progression through miRNA-411-dependent CDH2 inhibition. Am J Pathol. (2019) 189:2469–86. 10.1016/j.ajpath.2019.06.016 31476285

[46] Rivankar S. An overview of doxorubicin formulations in cancer therapy. J Cancer Res Ther. (2014) 10:853–8. 25579518 10.4103/0973-1482.139267

[47] Zhang S Liu X Bawa-Khalfe T Lu LS Lyu YL Liu LF Identification of the molecular basis of doxorubicin-induced cardiotoxicity. Nat Med. (2012) 18:1639–42. 10.1038/nm.2919 23104132

[48] Burridge PW Li YF Matsa E Wu H Ong SG Sharma A Human induced pluripotent stem cell-derived cardiomyocytes recapitulate the predilection of breast cancer patients to doxorubicin-induced cardiotoxicity. Nat Med. (2016) 22:547–56. 10.1038/nm.4087 27089514 PMC5086256

[49] Lu D Chatterjee S Xiao K Riedel I Huang CK Costa A A circular RNA derived from the insulin receptor locus protects against doxorubicin-induced cardiotoxicity. Eur Heart J. (2022) 43:4496–511. 10.1093/eurheartj/ehac337 35758064 PMC9637424

[50] Han D Wang Y Wang Y Dai X Zhou T Chen J The tumor-suppressive human circular RNA CircITCH sponges miR-330-5p to ameliorate doxorubicin-induced cardiotoxicity through upregulating SIRT6, survivin, and SERCA2a. Circ Res. (2020) 127:e108–25. 10.1161/CIRCRESAHA.119.316061 32392088

[51] Gupta SK Garg A Bär C Chatterjee S Foinquinos A Milting H Quaking inhibits doxorubicin-mediated cardiotoxicity through regulation of cardiac circular RNA expression. Circ Res. (2018) 122:246–54. 10.1161/CIRCRESAHA.117.311335 29133306 PMC5771684

[52] Du WW Yang W Chen Y Wu ZK Foster FS Yang Z Foxo3 circular RNA promotes cardiac senescence by modulating multiple factors associated with stress and senescence responses. Eur Heart J. (2017) 38:1402–12. 10.1093/eurheartj/ehw001 26873092

[53] Xu J Du WW Wu N Li F Li X Xie Y The circular RNA circNlgnmediates doxorubicin-inducedcardiac remodeling and fibrosis. Mol Ther Nucleic Acids. (2022) 28:175–89. 10.1016/j.omtn.2022.03.007 35402068 PMC8956965

[54] Wang X Cheng Z Xu J Feng M Zhang H Zhang L Circular RNA Arhgap12 modulates doxorubicin-induced cardiotoxicity by sponging miR-135a-5p. Life Sci. (2021) 265:118788. 10.1016/j.lfs.2020.118788 33245966

[55] Ji X Ding W Xu T Zheng X Zhang J Liu M MicroRNA-31-5p attenuates doxorubicin-induced cardiotoxicity via quaking and circular RNA Pan3. J Mol Cell Cardiol. (2020) 140:56–67. 10.1016/j.yjmcc.2020.02.009 32135167

[56] Zeng Y Du WW Wu Y Yang Z Awan FM Li X A circular RNA binds to and activates AKT phosphorylation and nuclear localization reducing apoptosis and enhancing cardiac repair. Theranostics. (2017) 7:3842–55. 10.7150/thno.19764 29109781 PMC5667408

[57] Han J Zhang L Hu L Yu H Xu F Yang B Circular RNA-expression profiling reveals a potential role of hsa_circ_0097435 in heart failure via sponging multiple MicroRNAs. Front Genet. (2020) 11:212. 10.3389/fgene.2020.00212 32211036 PMC7076158

[58] Li C Zhang L Bu X Wang J Li L Yang Z. Circ-LTBP1 is involved in doxorubicin-induced intracellular toxicity in cardiomyocytes via miR-107/ADCY1 signal. Mol Cell Biochem. (2022) 477:1127–38. 10.1007/s11010-022-04360-0 35076816

[59] Li B Cai X Wang Y Zhu H Zhang P Jiang P Circ-SKA3 enhances doxorubicin toxicity in AC16 cells through miR-1303/TLR4 axis. Int Heart J. (2021) 62:1112–23. 10.1536/ihj.20-809 34544967

[60] Xing X Tan Z Zhi X Sun H Yang J Li L Integrating analysis of circular RNA and mRNA expression profiles in doxorubicin induced cardiotoxicity mice. J Appl Toxicol. (2022) 42:793–805. 10.1002/jat.4257 34693535

[61] Jiang Y Shen X Dong C Zhi F Gao Y Shi C The whole transcriptome analysis and the circRNA-lncRNA network construction in arsenic trioxide-treated mice myocardium. Biomed Pharmacother. (2022) 151:113183. 10.1016/j.biopha.2022.113183 35676786

[62] Qiu Y Xie X Lin L. circFOXO3 protects cardiomyocytes against radiation induced cardiotoxicity. Mol Med Rep. (2021) 23:177. 10.3892/mmr.2020.11816 33398368

[63] Khan MA Reckman YJ Aufiero S van den Hoogenhof MM van der Made I Beqqali A RBM20 regulates circular RNA production from the titin gene. Circ Res. (2016) 119:996–1003. 10.1161/CIRCRESAHA.116.309568 27531932

[64] Tijsen AJ Cócera Ortega L Reckman YJ Zhang X van der Made I Aufiero S Li J Titin circular RNAs create a back-splice motif essential for SRSF10 splicing. Circulation. (2021) 143:1502–12. 10.1161/CIRCULATIONAHA.120.050455 33583186 PMC8032209

[65] Lu WY. Roles of the circular RNA circ-Foxo3 in breast cancer progression. Cell Cycle. (2017) 64:589–90. 10.1080/15384101.2017.1278935 PMC5397257 28278047

[66] Du WW Xu J Yang W Wu N Li F Zhou L A neuroligin isoform translated by circNlgn contributes to cardiac remodeling. Circ Res. (2021) 129:568–82. 10.1161/CIRCRESAHA.120.318364 34261347

[67] Zhu P Zhu X Wu J He L Lu T Wang Y IL-13 secreted by ILC2s promotes the self-renewal of intestinal stem cells through circular RNA circPan3. Nat Immunol. (2019) 20:183–94. 10.1038/s41590-018-0297-6 30643264

[68] Lin CC Hsu C Hsu CH Hsu WL Cheng AL Yang CH. Arsenic trioxide in patients with hepatocellular carcinoma: a phase II trial. Invest New Drugs. (2007) 25:77–84. 10.1007/s10637-006-9004-9 16937079

[69] Ohnishi K Yoshida H Shigeno K Nakamura S Fujisawa S Naito K Prolongation of the QT interval and ventricular tachycardia in patients treated with arsenic trioxide for acute promyelocytic leukemia. Ann Intern Med. (2000) 133:881–5. 10.7326/0003-4819-133-11-200012050-00012 11103058

[70] Cai BZ Meng FY Zhu SL Zhao J Liu JQ Liu CJ Arsenic trioxide induces the apoptosis in bone marrow mesenchymal stem cells by intracellular calcium signal and caspase-3 pathways. Toxicol Lett. (2010) 193:173–8. 10.1016/j.toxlet.2010.01.001 20079407

[71] Poller W Dimmeler S Heymans S Zeller T Haas J Karakas M Non-coding RNAs in cardiovascular diseases: diagnostic and therapeutic perspectives. Eur Heart J. (2018) 39:2704–16. 10.1093/eurheartj/ehx165 28430919 PMC6454570

[72] Zhang C Zhang B. RNA therapeutics: updates and future potential. Sci China Life Sci. (2023) 66:12–30. 10.1007/s11427-022-2171-2 36100838 PMC9470505

[73] Wesselhoeft RA Kowalski PS Anderson DG. Engineering circular RNA for potent and stable translation in eukaryotic cells. Nat Commun. (2018) 9:2629. 10.1038/s41467-018-05096-6 29980667 PMC6035260

[74] He AT Liu J Li F Yang BB. Targeting circular RNAs as a therapeutic approach: current strategies and challenges. Signal Transduct Target Ther. (2021) 6:185. 10.1038/s41392-021-00569-5 34016945 PMC8137869

[75] Qi LS Larson MH Gilbert LA Doudna JA Weissman JS Arkin AP Repurposing CRISPR as an RNA-guided platform for sequence-specific control of gene expression. Cell. (2013) 152:1173–83. 10.1016/j.cell.2013.02.022 23452860 PMC3664290

[76] Zhang Z Yang T Xiao J. Circular RNAs: promising biomarkers for human diseases. EBioMedicine. (2018) 34:267–74. 10.1016/j.ebiom.2018.07.036 30078734 PMC6116471

[77] Chen L Wang Y Lin J Song Z Wang Q Zhao W Exportin 4 depletion leads to nuclear accumulation of a subset of circular RNAs. Nat Commun. (2022) 13:5769. 10.1038/s41467-022-33356-z 36182935 PMC9526749

[78] Stewart M. Polyadenylation and nuclear export of mRNAs. J Biol Chem. (2019) 294:2977–87. 10.1074/jbc.REV118.005594 30683695 PMC6398137

[79] Hautbergue GM. RNA nuclear export: from neurological disorders to cancer. Adv Exp Med Biol. (2017) 1007:89–109. 10.1007/978-3-319-60733-7_6 28840554

